# Supplementary figures and images for: High prevalence of Schistosoma mansoni infection and stunting among school age children in communities along the Albert-Nile, Northern Uganda: A cross sectional study
Source: PLoS Negl Trop Dis. 2022 Jul 27;16(7):e0010570. doi: 10.1371/journal.pntd.0010570 (PMC9359559; doi:10.1371/journal.pntd.0010570)

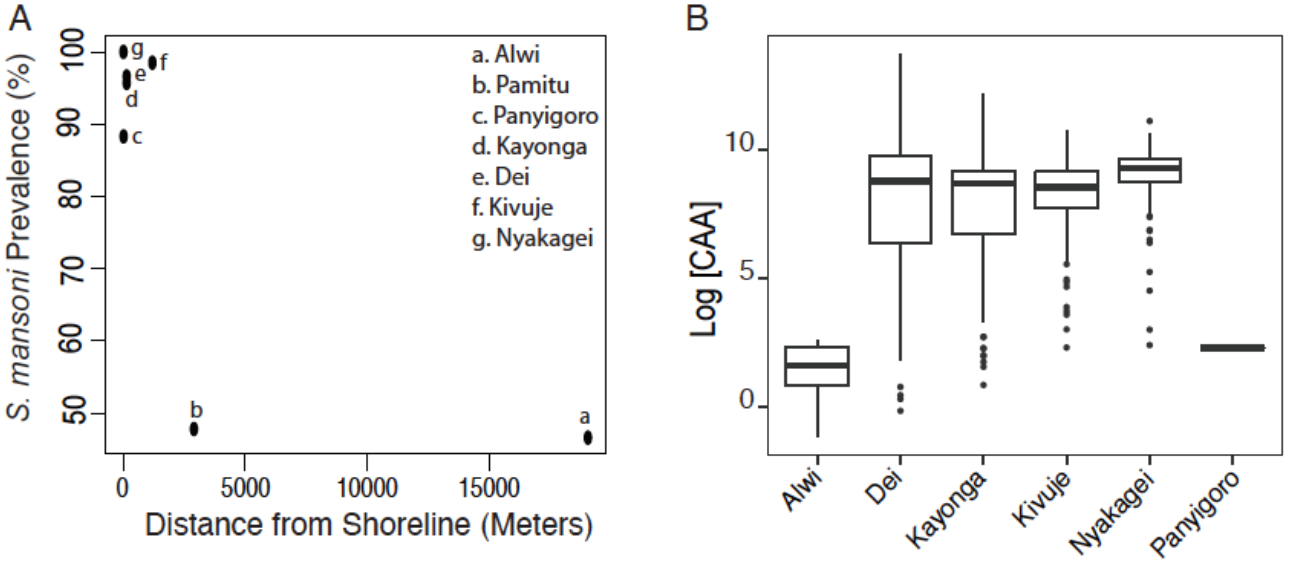

Supplement: S1 Fig — A. The prevalence of S. mansoni cases per site compared to their relative distance from the Albert Nile. The prevalence was determined by the POC-CCA screening test using the overall sample size of 914. B. The infection intensity per site as determined by the CAA test. (TIF) [file pntd.0010570.s001.tif]

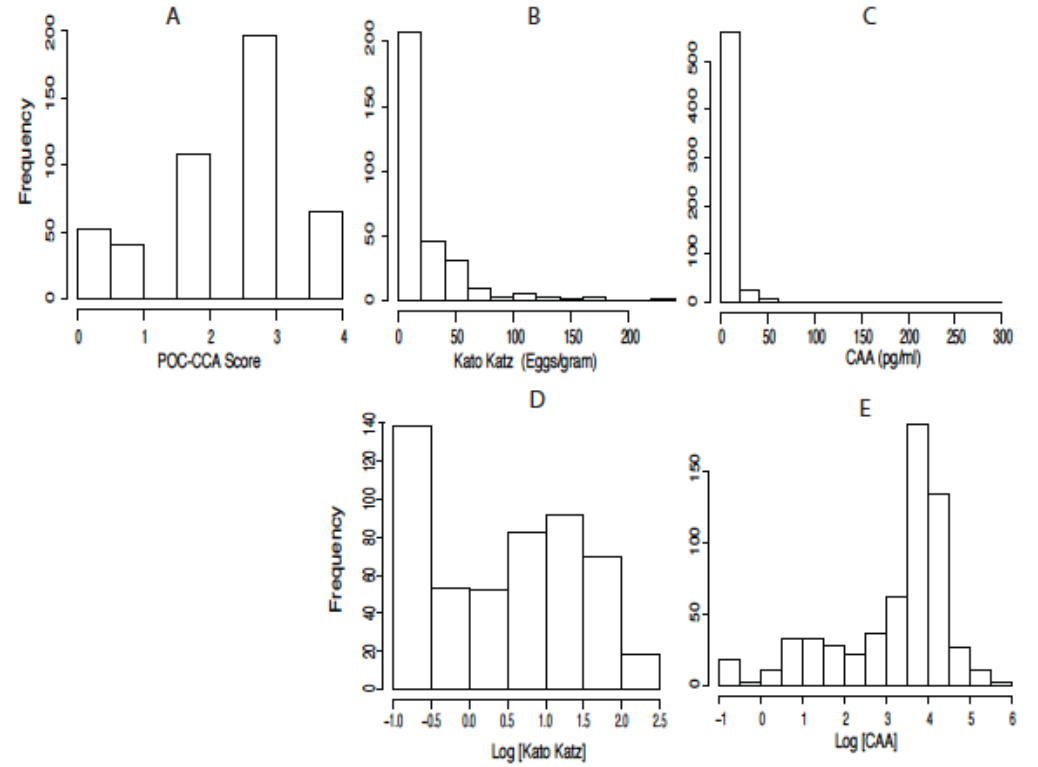

Supplement: S2 Fig — Frequency distribution of the POC-CCA (A) visual scores, Kato Katz (B) and CAA (C) intensities in the study samples. Log transformed KK (D) and CAA (E) intensities. (TIF) [file pntd.0010570.s002.tif]

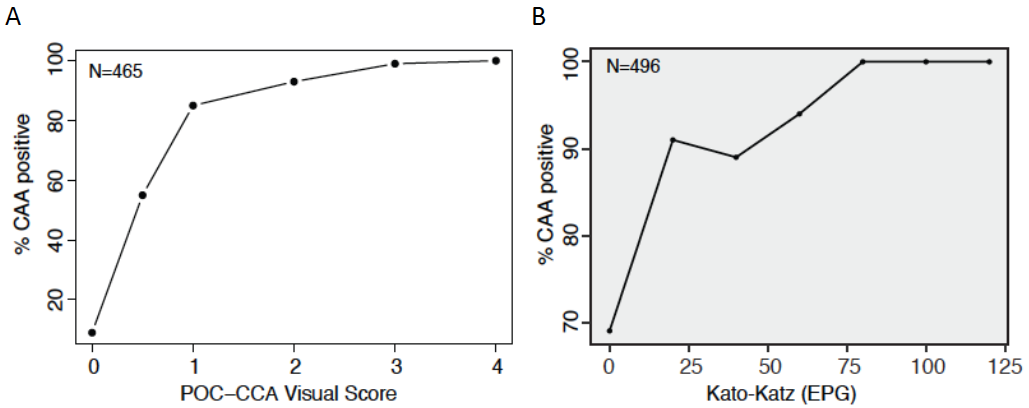

Supplement: S3 Fig — (TIF) [file pntd.0010570.s003.tif]

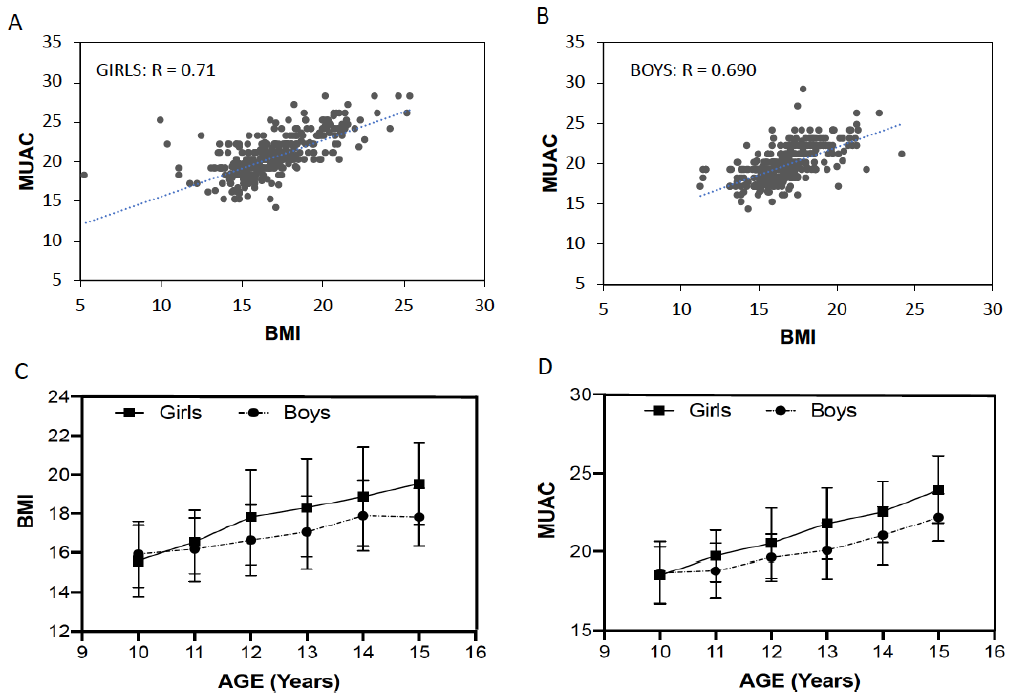

Supplement: S4 Fig — The bars in plots C and D indicate the standard deviations from the mean. (TIF) [file pntd.0010570.s004.tif]

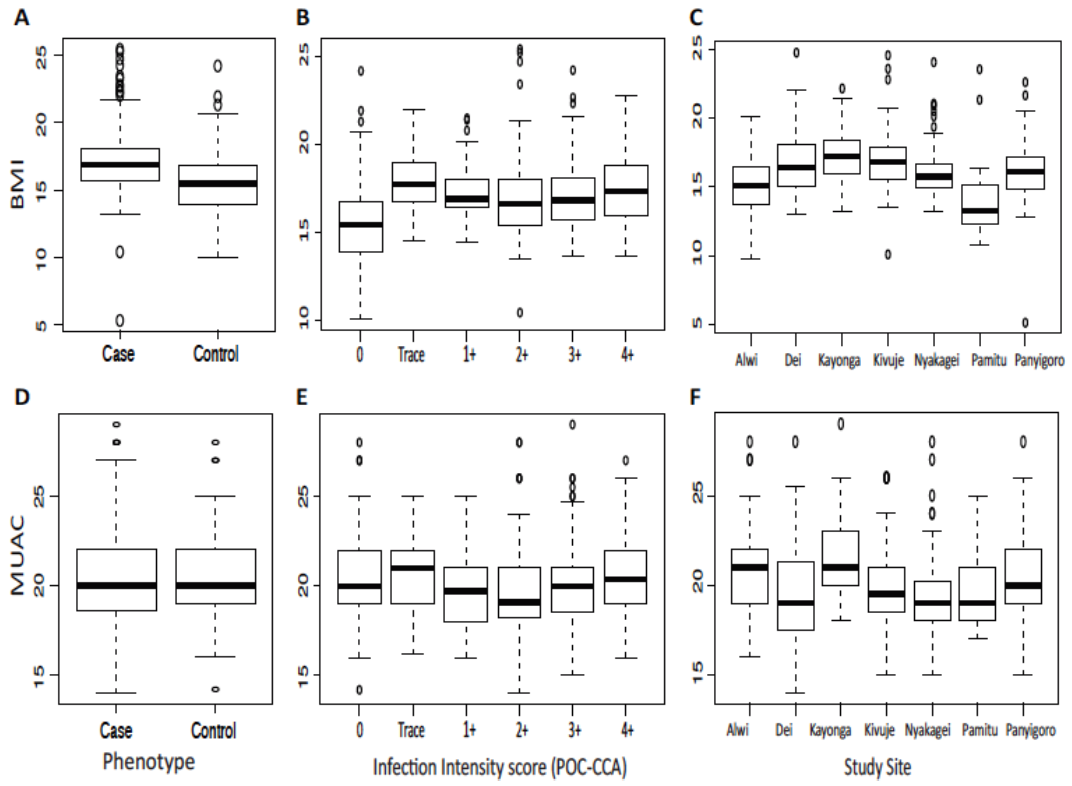

Supplement: S5 Fig — (TIF) [file pntd.0010570.s005.tif]

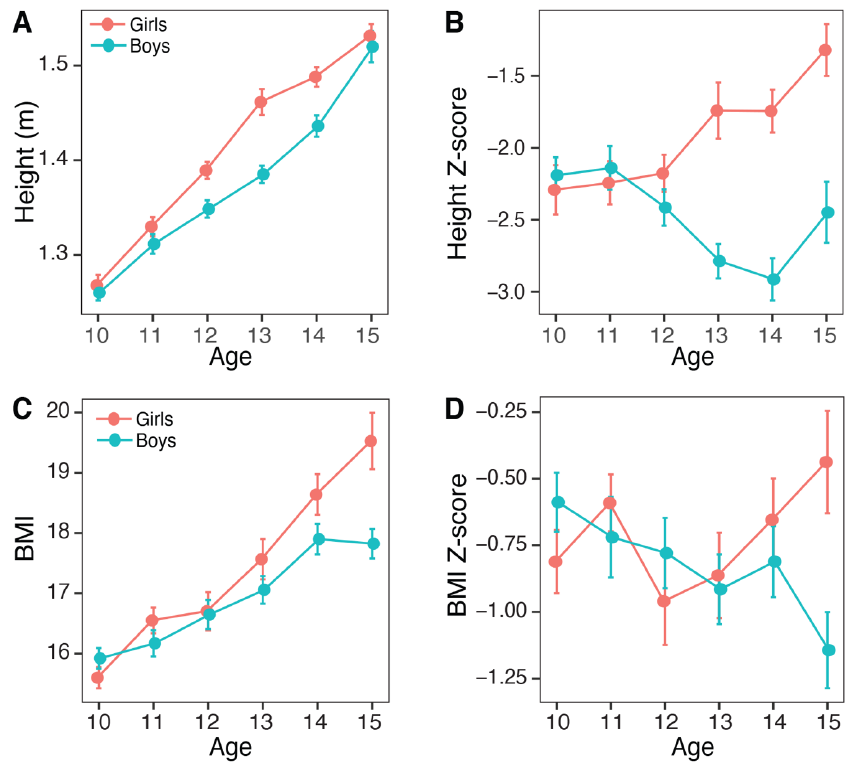

Supplement: S6 Fig — The age was compared with A. Height, B. Height Z-score, C. BMI and D. BMI Z-score. The vertical bars represent standard deviations from the mean. (TIF) [file pntd.0010570.s006.tif]

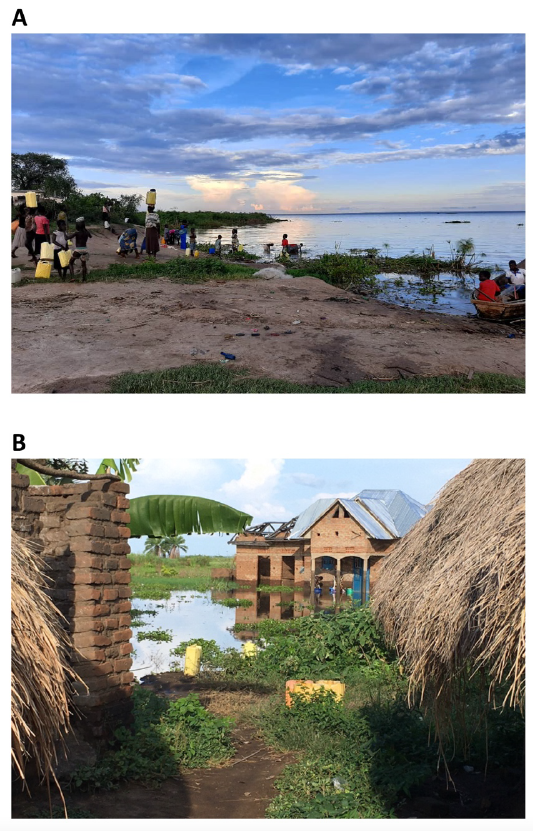

Supplement: S7 Fig — The author Julius Mulindwa was the photographer. A. Children and women taking water from the lake for household use. Picture was taken off the Dei sub county shoreline in November 2020. B. Submerged homes in Kayonga village, residents from this homestead were displaced. (TIF) [file pntd.0010570.s007.tif]

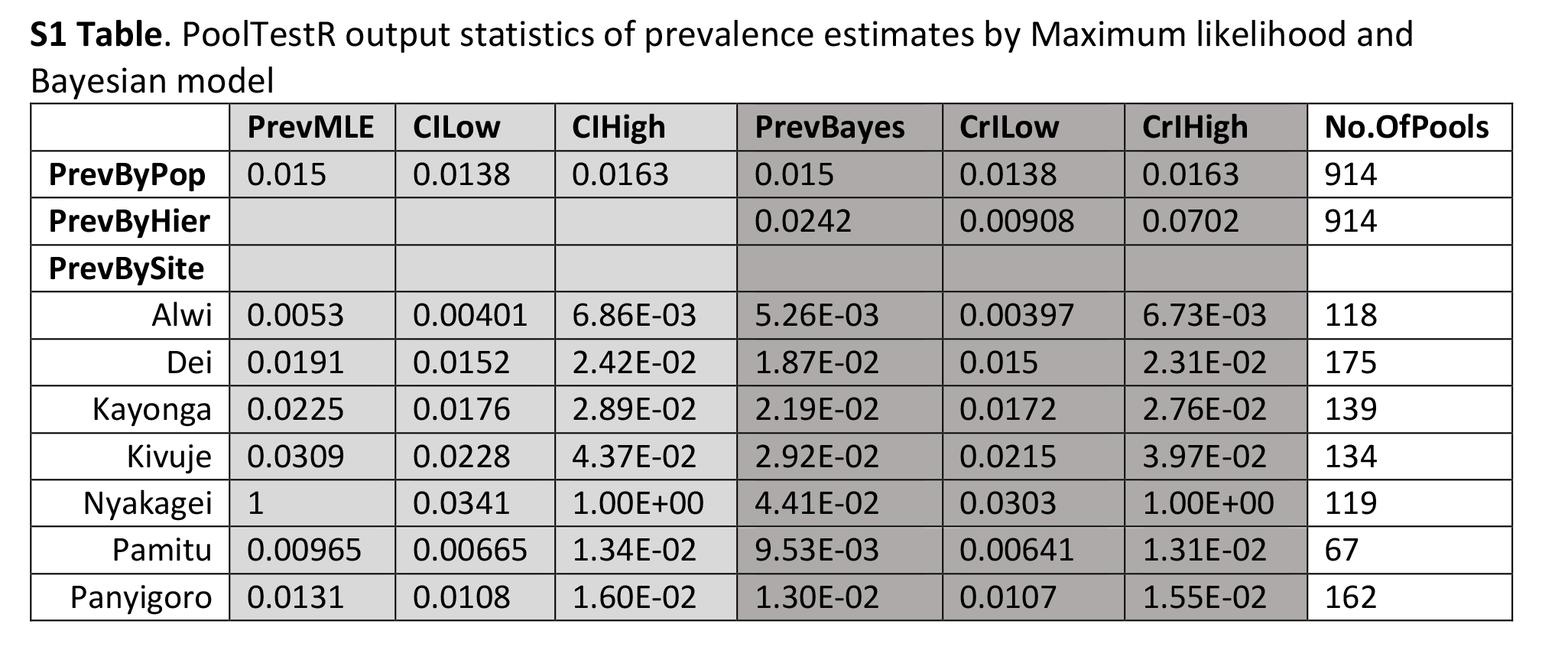

Supplement: S1 Table — (TIF) [file pntd.0010570.s008.tif]

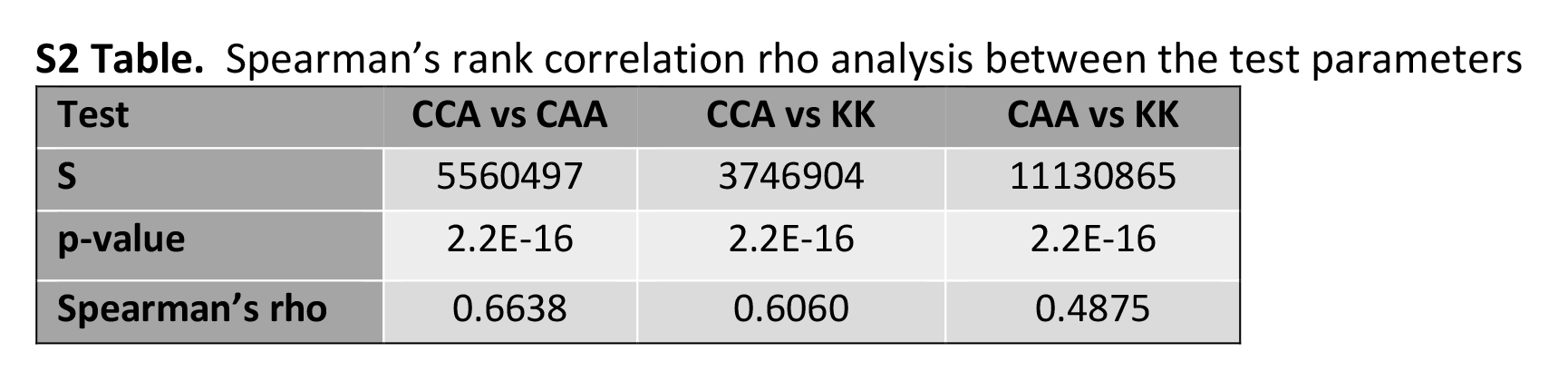

Supplement: S2 Table — (TIF) [file pntd.0010570.s009.tif]

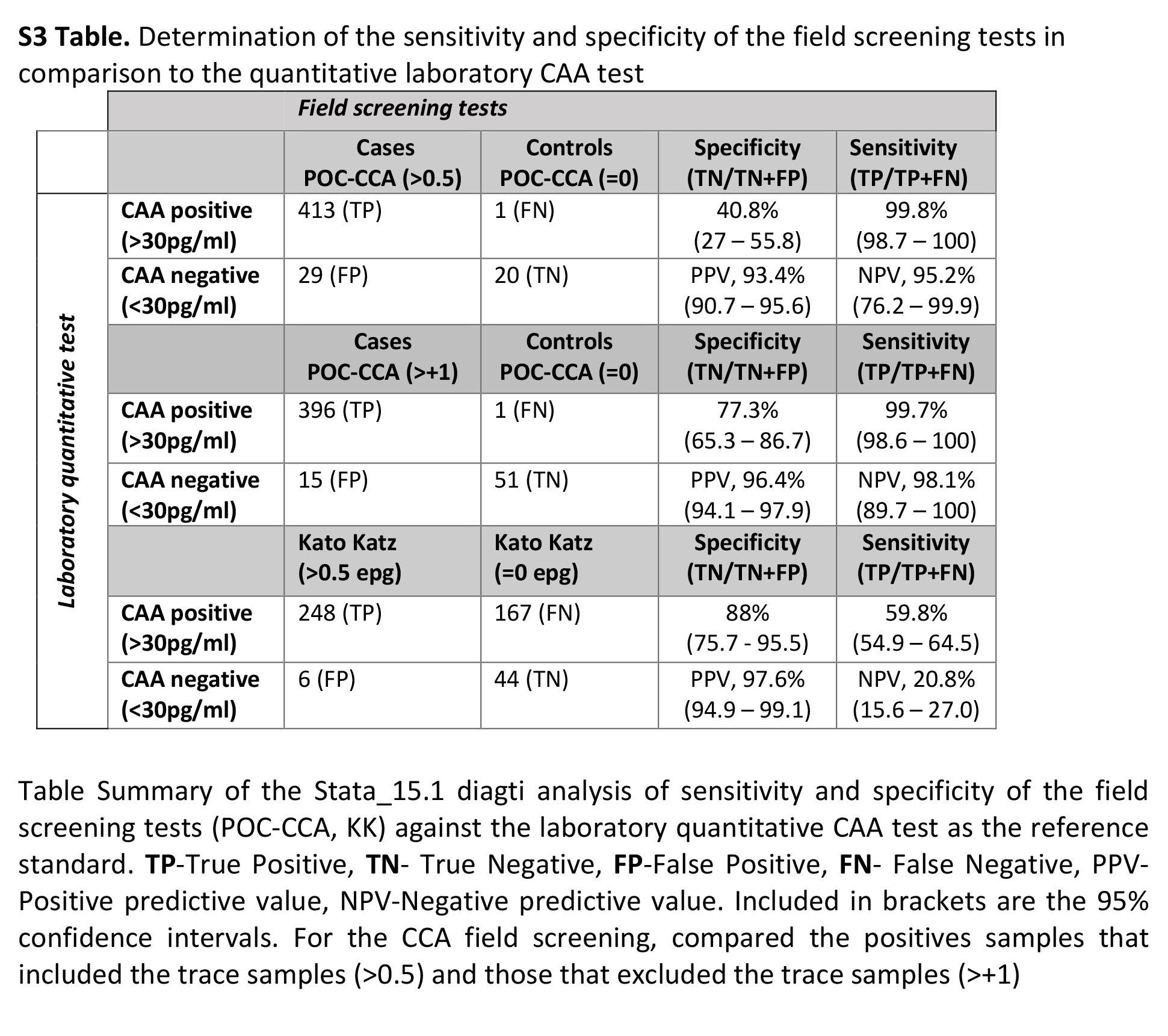

Supplement: S3 Table — (TIF) [file pntd.0010570.s010.tif]

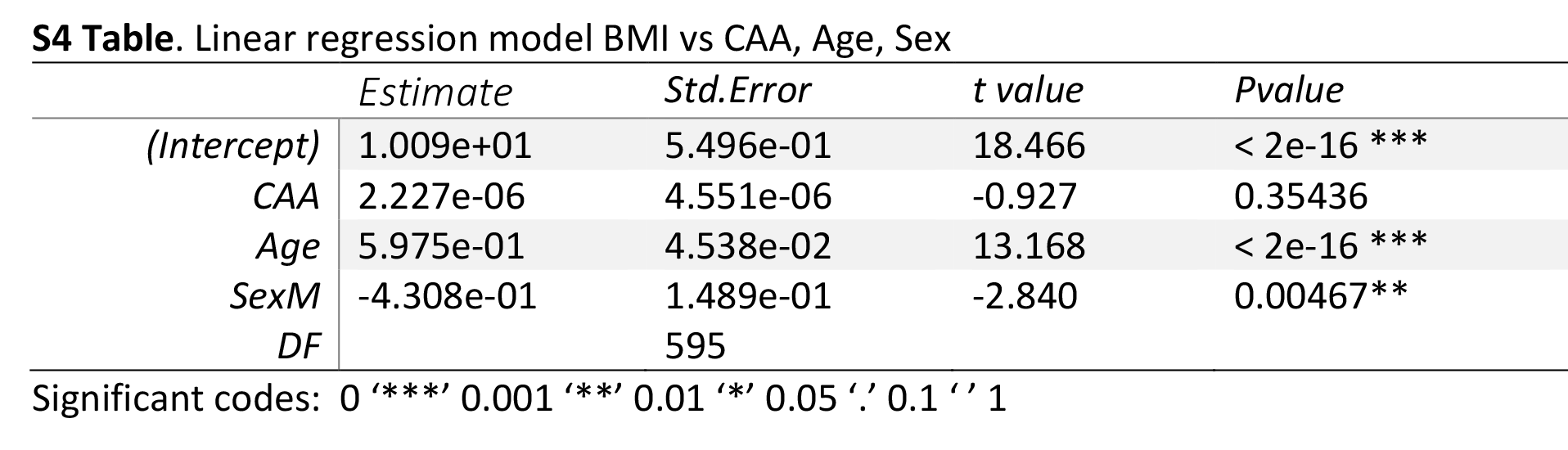

Supplement: S4 Table — (TIF) [file pntd.0010570.s011.tif]

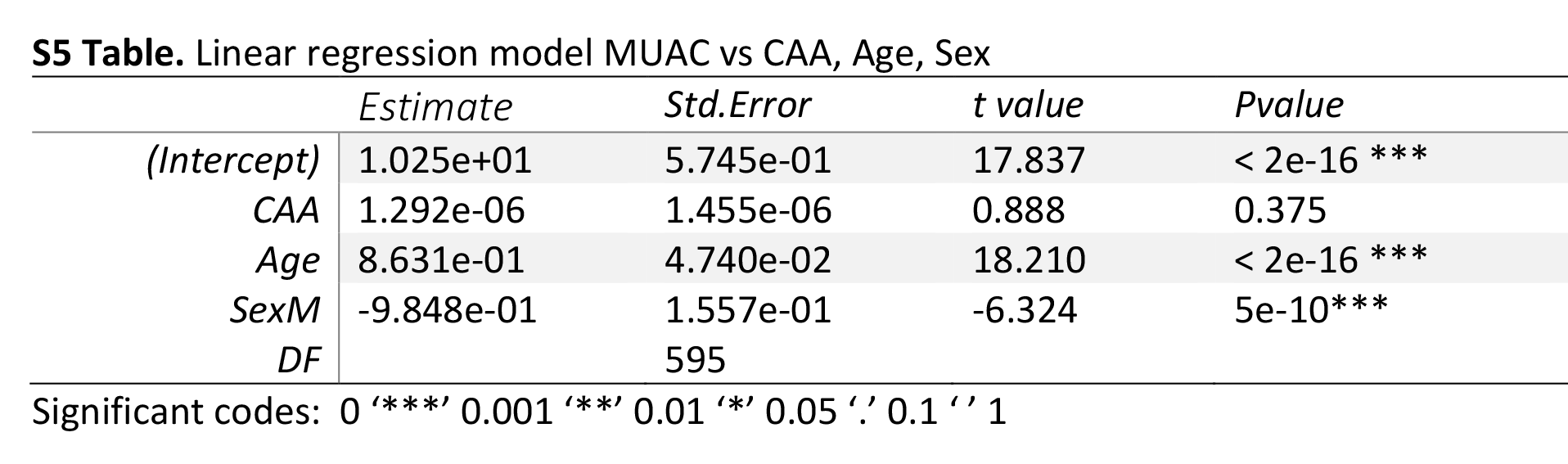

Supplement: S5 Table — (TIF) [file pntd.0010570.s012.tif]

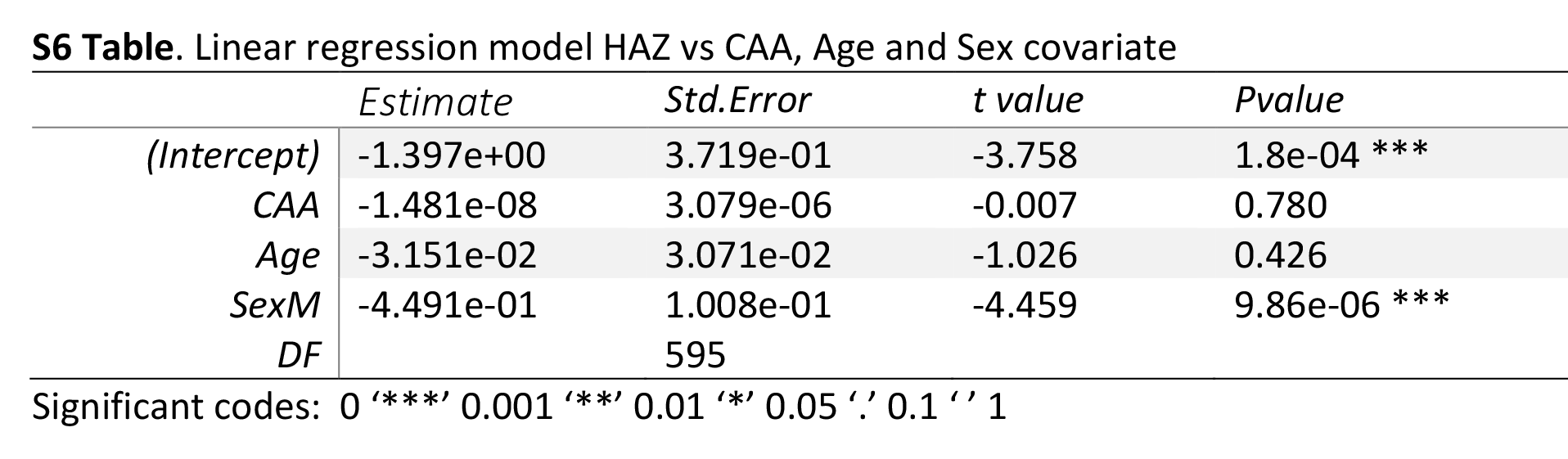

Supplement: S6 Table — (TIF) [file pntd.0010570.s013.tif]

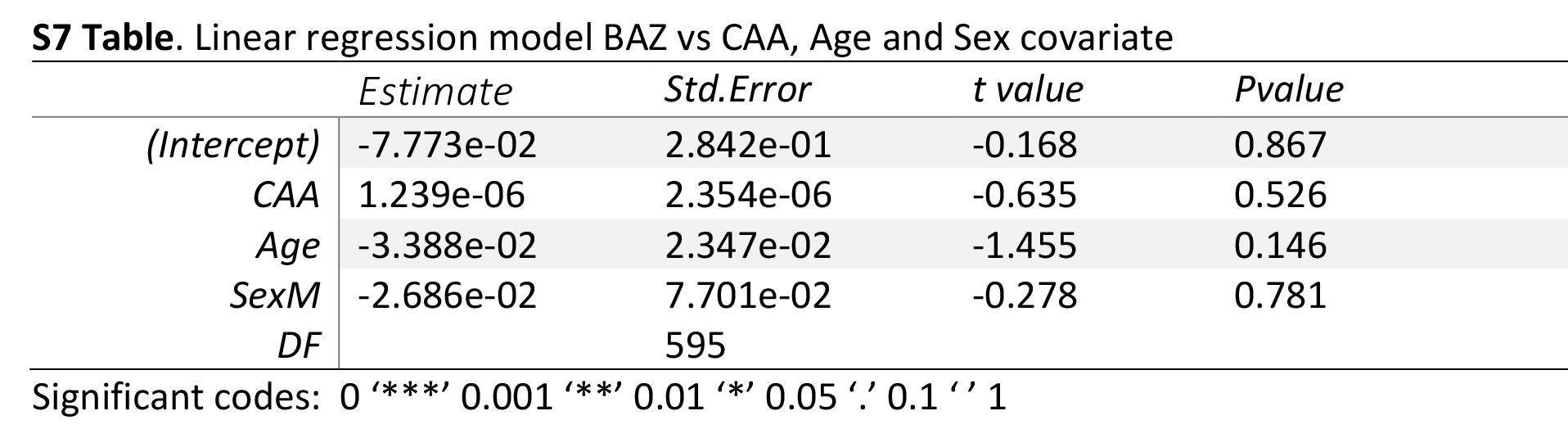

Supplement: S7 Table — (TIF) [file pntd.0010570.s014.tif]

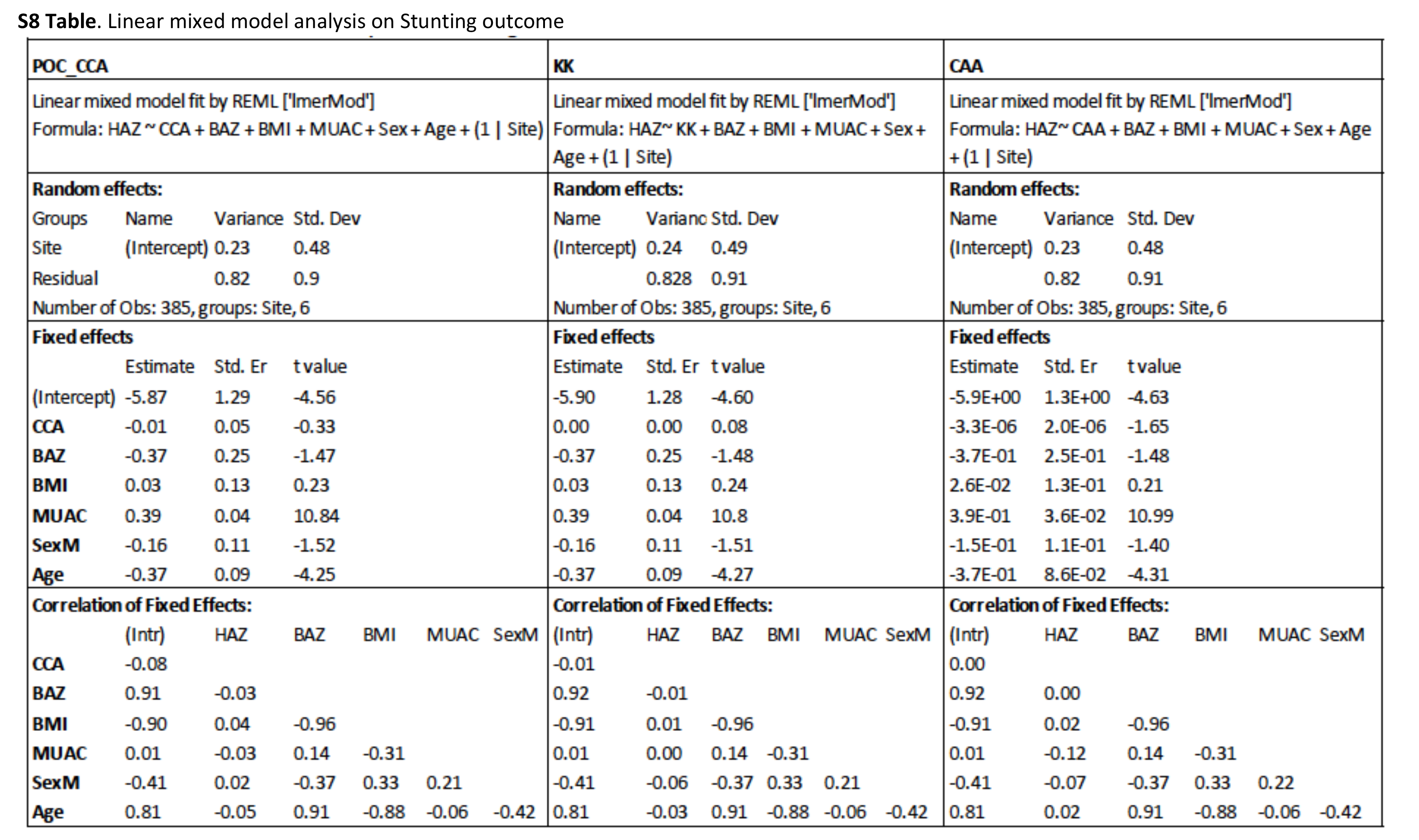

Supplement: S8 Table — (TIF) [file pntd.0010570.s015.tif]
