# Supplementary material for: High prevalence of Schistosoma mansoni infection and stunting among school age children in communities along the Albert-Nile, Northern Uganda: A cross sectional study
Source: PLoS Negl Trop Dis. 2022 Jul 27;16(7):e0010570. doi: 10.1371/journal.pntd.0010570 (PMC9359559; doi:10.1371/journal.pntd.0010570)
Supplement: Attachment — Submitted filename: Response_to_reviewers.docx [file pntd.0010570.s016.docx]

We thank the reviewers for their constructive comments which have enabled us to significantly revise the manuscript including additional statistical analysis of our data. Our responses to the specific reviewer comments are below:

Reviewer #1:

1. Title: add that this is a cross-sectional study: This has been added to the title

2. Abstract: the number of children examined for KK was < 914. Mention that Intensity of infection was assessed by POC-CCA and KK as well as CAA. Mention that KK intensity was also correlated with CAA and with CCA intensity. This statement has been added to the text

3. Author summary: intensities of infections were light, according to WHO guidelines. This statement has been added to the text

4. Introduction:

5. Line 106: strictly speaking a viable egg is one that goes on to develop into the next stage. KK enables to detects eggs, but it cannot distinguish between viable and non-viable eggs. I suggest that the word “viable” is removed. The word ‘viable’ has been removed

6. Lines 116-118: pros and cons were mentioned for KK and CAA but not for POC-CCA. It might be worth mentioning current concerns associated with POC-CCA (e.g. Peralta and Cavalcanti 2018). The statement and citation “although misdiagnosis has been observed with trace results of the POC-CAA (Peralta and Cavalcanti 2018)” has been added to the text

7. Lines 101 and 124 report different ages from school aged children (5-15) and (6-15).

The ages in line 124 have been corrected to 5-15

8. Lines 125-128: the relationship between schistosomiasis infection and nutritional status and cognitive impairment has not been firmly stablished yet (e.g. Welch et al. 2017). I suggest that this is acknowledged. This statement has been added and cited in the text

9. Line 130: as this is a cross-sectional study, causal relationships cannot be ascertained. I suggest that the word “affect” is replaced with “association”. The word “effect” has been replaced by “association with”.

10. Line 155: are the four sub-counties visited representative of the whole district? E.g. How many sub-counties are there in Pakwach? If there are more than 4 sub-counties, how did they choose to survey these 4 sub-counties?

Pakwach district consists of 6 sub counties of which 4 were chosen for the survey based on their prior endemic status Exum et al., 2018 and being transmission hot spots (Communication from Vector control division, Ministry of Health Uganda).

11. Line 157: are the children representative of the sub-counties? E.g. What was the sampling frame for the collection points? How were the collection points selected? Is there a possibility that some children in the district could not reach the collection points and that the results could be biased to reflect the prevalence around the collection points rather than the prevalence of the whole district? The initial sampling was meant to be from schools however due to the Covid19 pandemic these schools were closed. Therefore, the collection points were the schools (and health centre in some cases) where the children from the surrounding villages could gather and observe COVID19 SOPS. As shown on the map (Figure 1), the collection points were located in 4 sub counties which were on average 20km apart except for those in Panyimur subcountry (Dei, Kayonga, Nyakagei). Given the time of the survey was during the Covid19 lockdown (October-November 2020), not all the children could make it to the collection points however the Village health teams (VHTs) community out-reach ensured a good turn up of the children to the sites.

Target were the villages closest to the lake (hotspots) where the prevalence of *S. mansoni* has previously been reported to be high (Reports from Vector Control Division, Ministry of Health, Uganda).

12. Line 164: it was not clear upon first read of the paper that the survey comprised two stages (a screening phase and the main survey phase). I suggest that this is clearly outlined before explaining the screening process. A statement has been added at the end of the study area and population section, “The survey consists of two main phases namely, the screening of the children for schistosomiasis and the recruitment of the eligible participants for sample collection”. I suggest that the reasons why the children were asked to return the next day with the parents are detailed (i.e. to carry out main survey?). The statement has been edited at line 171 as, “Those children that complied and brought the urine for testing were requested to return the following day with their parents for the recruitment exercise into the main survey”. Would having to return the next day have impacted the representativeness of the sample? Is it possible that those children living far from school could not return to the collection site on the second day? Indeed some children were not able to return especially if their parents or guardians were not available, a key criteria for consent as indicated by the numbers that were recruited.

13. Line 168 indicates that POC-CAA was assessed for all 10–15-year-old children. Line 183 indicates that participants were randomly selected. Does this mean that all the children that turned up in day one were surveyed and only some of them were asked to return the next day? How was the required sample size estimated and how was the random selection conducted? The children were purposively selected, the word “Random” has been deleted from the statement. Children in the 10-15 age bracket that had been screened the previous day and returned with their Parent/ guardian were considered. Sample size calculation was done using Kish and Leslie’s formula and this has been included in the text.

14. Line 185: was POC-CCA measured on day 1 while CAA, KK and anthropometric measurements were taken on day 2? The KK and anthropometric measurements were done on day 2, whereas the CAA was carried out on the frozen plasma samples at a later date after the field survey. This has been mentioned on line 170 and has been modified to read as, “Those children that complied and brought the urine for testing were requested to return the following day with their parents for the recruitment exercise into the main survey”

15. Lines 243-4 indicated that 80% of the POC-CCA screened children assented to participate. It is ambiguous whether consent and assent for POC-CCA had been granted or not. This needs to be specified. The parent/guardian’s consent and child’s assent to participate in the study was obtained on day 2 following the screening on day 1.

16. Line 209: do they know the precision of the weighing scale? How was aged obtained (how reliable is this variable)? The weigh scale was calibrated to ensure precision. The age was obtained from the parent/guardian through stating the date of birth or year of birth.

17. Lines 233-4: the way that overall prevalence (and district summary statistics) is calculated gives more weight to those locations that had more participants and does not take into account the hierarchical structure of the survey design. A way to overcome this could be to estimate prevalence using random effects for collection sites, e.g. with R package lme4. The R “survey” package can also be used. To estimate the prevalence from our sample data, we adjusted for hierarchical sampling structure using the PoolTestR package. This has been included in the methodology Statistical analysis section. The prevalence estimates from this analysis have been added as a supplementary table S1.

A statement asserting these prevalence estimates has been added to the text.

18. Lines 235-238: t-tests, ANOVA and linear regression are valid when the underlying assumptions hold. Have the authors checked that this is the case? Spearman correlation was carried out but this method is not listed in this section. The authors use “95% confidence level (CL)” in Table 2, line 280, and other places. What is 95% CL? How should it be interpreted? They have not defined what level they consider as significant. E.g. is it 0.05 or 0.1? I suggest that p-values smaller than 0.001 are reported as “<0.001” instead of reporting all the decimal figures. We have rewritten the statistical analysis section and included the distribution and data transformation methods, the analysis tests carried out including the tools used (including Spearman’s) and threshold levels of significance.

19. To avoid misinterpretations I suggest that the authors define what they mean by “phenotype”, “case” and “control”. The term phenotype has been removed from the text body. The case and control definitions have been included in the text under the section of “Screening for Schistosomiasis”

Reviewer #2: Overall the study design was appropriate and the study population has been clearly described.

However, addressing the balance of the article across the stated objectives would strengthen the work.

For example, the authors begin with 'Efforts to control schistosomiasis infection in Uganda have been greatly affected by the limited knowledge of the extent of infection, impact of disease on child growth and the inconsistency in delivery of treatment through mass drug administration.'

Nevertheless, the majority of the article compares the relative merits of screening tools for S. mansoni. While this is valuable, this aim needs to be better explicated in the summary and introduction and the implications better detailed in the discussion/conclusion. A statement comparing the sensitivity outcome of the screening methods of POC-CCA and Kato Katz has been included in the summary and also indicated the evaluation and comparison of CCA, KK and CAA techniques in the aim of the study under the introduction section. Details of the comparison of the tests with regards to screening in a rural field setting had been included in the discussion section.

The authors found an 85% prevalence of S.mansoni among the children involved. Yet these children were apparently participating in an annual MDA programme. Further information about the MDA programme, the uptake among the children and indeed, the potential reasons for the failings of this public health intervention would be of interest to both general readers and policy makers. This survey was carried out independently of the MDA. The MDA had previously been done in 2018 and most recently in the month of December 2020, whereas this survey was done in the October and November 2020. A statement relating the possible high prevalence and having missed MDA in 2019 has been added to the discussion, “The high prevalence of schistosomiasis in these sites was probably due to the inconsistence in the MDA, which had been last carried out in the year 2018 in this region (Communication from District Health Office, Pakwach) and thereafter in December 2020 (before this study survey)”.

--------------------

Results

-Does the analysis presented match the analysis plan?

-Are the results clearly and completely presented?

-Are the figures (Tables, Images) of sufficient quality for clarity?

Reviewer #1: 20. Line 242: should it say 4 sub-counties? This has been changed to 4

21. Line 248: consider including a flow diagram with the number of children that were assessed for each diagnostic test: e.g. 914 children that provided POC-CCA samples, 727 children were recruited for further studies of which 554 were analysed for KK and 600 for CAA; 387 provided samples for all three tests. A flow diagram has been created and included as Figure 1b.

22. Lines 249-255 are methods rather than results and not all of them apply to this study. I suggest that the authors consider removing these lines. This paragraph has been removed from the text. It might be worth mentioning the TrypanoGEN+ study in the introduction and contextualise this manuscript within the TrypanoGEN+ study. The statement “This study was conducted as part of the TrypanoGEN+ consortium projects (<http://trypanogen.net)>”, has been added at the end of the introduction.

23. Lines 258 and 262: as already mentioned, I suggest that the authors consider using hierarchical modelling to estimate population prevalence. Hierarchical modelling has been carried using the R package PoolTestR, the prevalence estimates incorporated and a Table S1 included.

24. Line 260: do the authors mean 4 to 15 or 10 to 15? It is 10-15, this has been corrected

25. Line 261: epiR was used to estimate the confidence interval for prevalence but the authors do not give details about the statistical method used in the calculation. For example, was it calculated using Wilson’s method or the exact method? This statement has been revised to include the method as, “by using epiR exact method, the estimated prevalence of schistosomiasis in this population was 85% (95% CI 83.2 - 87.87).”

26. Intensity of infection:

a) As intensity was measured in three different ways, and to avoid confusion, when mentioning intensity, it would be useful to refer to the method used (i.e. kk, POC-CCA or CAA) The method used for intensity measurements has been clearly referred to in line with the context

b) Check that the statistical tests used (t-test and ANOVA) can be used i.e. that the assumptions hold. If they don’t hold then consider transforming the data or using non-parametric tests. The data distribution was checked using R gladder package for which Log transformation of the KK and CAA data has now been used on the parametric tests shown in Table 2

c) POC-CCA scores might be better assessed with chi squared tests than by comparing means. A chi squared test was carried out and the data has been recorded in Table 2

d) When reporting ratios, also include their confidence intervals (lines 291 and 304)

e) The p-value in line 303 of 0.096 is considered to be significant. Stablish in the methods the p-value threshold that will be used throughout the paper. This paragraph has been re-written following data transformation

f) Consider applying hierarchical multivariable regression

Hierachical multivariable regression analysis has been used when comparing all the variables of infection intensity and nutrition status for which we have used the R packages lmer and a table S8 included.

Correlation:

27. Line 318: although the correlation coefficient of 0.48 is the lowest of the three reported, it is technically not “low”. We have revised the term to “relatively low” to rationalise that it is not technically low

Sensitivity/specificity:

28. Note that 6 KK positive cases were CAA negative. Comment on the implications of this with respect to the estimated sensitivity/specificity. A statement has been added, “the 6 positive cases that were negative by CAA did not greatly affect the specificity of the test”

29. Report confidence intervals for sensitivity and specificity estimates. The 95% confidence intervals have been added to the text

30. Line 346: says in the study population. However, the reported value is a sample result. This statement has been corrected to indicate the study sample population

31. Line 336 refers to POC_CCA intensity (Fig S5b). However, the multivariable analysis was done on CAA intensity. I suggest that these tests are done on the same intensity results, either POC-CCA or CAA. This has been validated using the linear mixed multivariable analysis

32. Line 340: for completeness, it might be good to do a multivariable test on MUAC.

The MUAC was included in the linear regression (Table S5) and mixed model analysis (Table S8)

Tables and figures:

37. Table 2 caption: “Summary descriptive statistics and between groups comparisons for the POC-CCA, Kato Katz and CAA test”. This has been corrected

38. Figure 1: would it be possible to add the sub-county names and border lines? Subcounty borders have been added to the figure

39. Figure 2: “Schistosoma mansoni” This has been corrected

40. Figure 3A. Numbers add up to 83%. Shouldn’t this be 100%? The Numbers correspond to the participants with positive visual scores (non-Zero scores)

41. Figure 4: as no correlations are reported on the graphs, would it be more appropriate to label this graph as “relationship between…” instead of “correlation”? I would suggest changing “absolute measured values” to “original scale values”. These changes have been implemented in the text

Supplementary data:

42. Figure 1: mention what test was used to determine prevalence and the overall sample size. A: Make “S. mansoni” on the y-axis italics. B consider using log-scale for the y-axis values. These changes have been added to the supplementary data

43. Figure S3. What would a similar plot for CAA and KK look like? A plot for %CAA vs KK has been added to supplementary data (Figure S3B)

44. Figure S4. What do the vertical bars represent? Are they confidence intervals? The vertical bars are standard deviations from the mean and this has been included in the figure legend.

45. Figure S6. What do the vertical bars represent? The bars represent standard deviations from the mean and this has been indicated in the figure legend

46. Tables S4 to S6: Is the first row in each table the intercept? Would it be possible for the tables to include the regression coefficients? The regression coefficients have been included to the tables

Reviewer #2: See above.

The authors findings regarding the varying prevalence of Schistosomiasis among children living in a radius from the Albert-Nile is important. Yet little detail is provided as to potential reasons for these differences or indeed, if particular sub-populations of children had greater/lower risk i.e. presumably children who have consistently attended school even in high risk areas will have had lower exposure rates?

In the discussion we mentioned the potential factors for the varying prevalence, which have also been observed in other studies. The close proximity to the lake and exposure to water contaminated with infective cercariae predisposes children to Schistosomiasis

The analysis in relation to child stunting while interesting, lacks nuance. Identifying the potential interactions between the drivers of child stunting and Schistosome infection across this population would enhance this impact and usefulness of this work. A follow up study on dietary intake and environmental exposure in relation to stunting and knowledge attitude and practices in this Schistosomiasis foci is underway and therefore not part of this manuscript

Equally, when exploring child stunting, the KK results were not presented. Again, following through on the current frame of the article around the comparison of the three screening tools, presenting a more complete analysis here would be of interest. The KK effect on stunting outcome has now been reported in the linear mixed model results. The inclusion of the multivariable linear mixed models have endevoured to address the comparison of the three methods on the observed nutritional status measure of stunting and wasting.

--------------------
