# Supplementary material for: High prevalence of Schistosoma mansoni infection and stunting among school age children in communities along the Albert-Nile, Northern Uganda: A cross sectional study
Source: PLoS Negl Trop Dis. 2022 Jul 27;16(7):e0010570. doi: 10.1371/journal.pntd.0010570 (PMC9359559; doi:10.1371/journal.pntd.0010570)
Supplement: Attachment — Submitted filename: Response_to_reviewer_comments_V6.docx [file pntd.0010570.s017.docx]

Dear Dr. Matovu,
 
Thank you very much for submitting your revised manuscript "High levels of Schistosoma mansoni infection and stunting among school age children in communities along the Albert-Nile, Northern Uganda: a cross sectional study" for consideration at PLOS Neglected Tropical Diseases. As with all papers reviewed by the journal, your manuscript was reviewed by members of the editorial board and by independent reviewers. In light of the reviews (below this email), whilst we appreciate that improvements to the text have been made, we would like to invite the further resubmission of a significantly-revised version that takes into account the reviewers' comments. In particular, please ensure you carefully respond to and accommodate fully each of the highly valid and extremely useful further comments provided by referee 1 - which includes those highlighting where your responses to date (such as regarding the statistical analyses used here) have not sufficiently rectified the issues highlighted before.

 
We cannot make any decision about publication until we have seen the revised manuscript and your detailed response to the reviewers' comments. Your revised manuscript is also likely to be sent to reviewers for further evaluation. However, please also note that, in order to minimize the risk of reviewer fatigue, that this will be the final opportunity to resubmit here.
 
When you are ready to resubmit, please upload the following:

[1] A letter containing a detailed list of your responses to the review comments and a description of the changes you have made in the manuscript. Please note while forming your response, if your article is accepted, you may have the opportunity to make the peer review history publicly available. The record will include editor decision letters (with reviews) and your responses to reviewer comments. If eligible, we will contact you to opt in or out.

[2] Two versions of the revised manuscript: one with either highlights or tracked changes denoting where the text has been changed; the other a clean version (uploaded as the manuscript file). 

Important additional instructions are given below your reviewer comments.

Please prepare and submit your revised manuscript within 60 days. If you anticipate any delay, please let us know the expected resubmission date by replying to this email. Please note that revised manuscripts received after the 60-day due date may require evaluation and peer review similar to newly submitted manuscripts.
 
Thank you again for your submission. We hope that our editorial process has been constructive so far, and we welcome your feedback at any time. Please don't hesitate to contact us if you have any questions or comments.
 
Sincerely,

JOANNE P. WEBSTER
Associate Editor
PLOS Neglected Tropical Diseases

Christine Budke
Deputy Editor
PLOS Neglected Tropical Diseases

***********************

Reviewer's Responses to Questions

**Key Review Criteria Required for Acceptance?**


As you describe the new analyses required for acceptance, please consider the following:

**Methods**

-Are the objectives of the study clearly articulated with a clear testable hypothesis stated?
-Is the study design appropriate to address the stated objectives?
-Is the population clearly described and appropriate for the hypothesis being tested?
-Is the sample size sufficient to ensure adequate power to address the hypothesis being tested?
-Were correct statistical analysis used to support conclusions?
-Are there concerns about ethical or regulatory requirements being met?


**Reviewer #1:**

**-Are the objectives of the study clearly articulated with a clear testable hypothesis stated?**
Some objectives are clearly articulated but (at least) the following are missing: comparison of hotspot sites with non-hotspot sites (lines 177-179), assessment of differences in prevalence and intensity between, sexes, ages and locations. The following statement has been added to the aim line 141 “We compared the hotspots with non-hotspot sites and assessed the prevalence and intensity of S. mansoni infection between sexes, ages and locations.”
Introduction: not enough is said about children’s nutrition status in Uganda. The following paragraph has been added to the introduction “In Uganda, 30% of the children under five years are reported to be stunted, many of whom are in rural areas. Among the drivers of malnutrition in these areas has been the lack of access to clean water, poor sanitation, poor feeding practices and high disease burden (UBOS, 2016).”

**-Is the study design appropriate to address the stated objectives?**
Yes, the study design is appropriate.

**-Is the population clearly described and appropriate for the hypothesis being tested?**
There is some ambiguity with respect to what the study population is. Is it “primary school going children living in recognised schistosomiasis high transmission hotspots in the West Nile sub-region district 166 of Pakwach“ (lines 164-165)? If so, then the extrapolation of prevalence to the whole district would be inaccurate (lines 22-293), as the study was not designed to estimate district prevalence. The prevalence estimates of the whole district have been removed. As well as this, if the population of interest was that of the hotspots, shouldn’t the two sites that were located away from the river have been excluded from the analyses? The two sites located away from the river highlight the spatial variation in prevalence (the sites further from the river also report cases) and effect of even a fairly small distance from Nile on the prevalence. We therefore feel that they should be included in the analysis.
It might be useful to define “hotspot” the first time this word is used (line 140).

The definition of “hot spot” has been included in the introduction section as a statement, “In Uganda, some villages have had persistent high intensity and/or prevalence of schistosomiasis despite repeated MDA and these are referred to as persistent hotspots [18]”

**-Is the sample size sufficient to ensure adequate power to address the hypothesis being tested?**
Sample size, ignoring non-response rate, was 97 (not reported by the authors). This has been addressed using the Kish and Leslie sample size calculation (line 186) with a 10% adjustment for non-response (line 189), we estimated the sample size required to be 128 children per site (line 190). Two sites had fewer participants than required (Pamitu 32, Alwi 62). No mention of this is made in the study. The following statement has been added under the Results, Participant recruitment and sample collection, “On comparing the number of children who consented to take part in the study verses those that were screened (727/914), we observed that Alwi and Pamitu had lowest percentage recruitment of 52% (62/118) and 47% (32/67) respectively.”

The notation on the formula for sample size is confusing: ensure the alpha is subscripted (line 186) and remove “P<0.05” from line 188. The formula has been edited,we have removed the alpha (this does not affect the interpretation) and also removed the “(p<0.05)”.

**-Were correct statistical analysis used to support conclusions?**
The statistical analyses used were not properly described. Please present a clear analysis plan, with sufficient description and justification of the methods used. In order to show the statistical analysis used for the study, a statement added under the section on statistical analysis, “The data collected on recruitment of the children which included the disease detection and quantification (POC-CCA scores, Kato Katz and CAA), anthropometric measurements (Height, Weight, Age and Sex) and the site of data collection were used for the quantitative analysis of Schistosomiasis prevalence, infection intensity and nutritional status of the children.”

The statistical analysis section has been rewritten with clear description and justification of methods used.
The authors are not consistent in their application of hierarchical modelling across tests.
Line 260: do the authors mean “overall” instead of “population prevalence”? Use of overall instead of population prevalence
Line 261: do the authors mean “point” instead of “sample”? Use point instead of sample
Lines 261-264 need clarification. For instance, why do the authors estimate confidence intervals and credible intervals? etc. These are the outputs from the PoolTestR algorithm when estimating the prevalence using the maximum likelihood (confidence interval) and Bayesian model (credible intervals), and these take into account the site populations supplementary table S1.
Lines 268-269 also need clarification. For example, what is being achieved by this technique? The raw data was transformed to follow close to normal distribution in order to analyse for linear distribution between variables (KK vs CAA). This is the reason why the distribution was checked and parameters transformed to Log scale as the best normalisation model.
There is no information in the statistical methods section about how POC-CCA sensitivity and specificity were estimated. For example, using CCA as a gold standard; how were the confidence intervals estimated? Mention the limitations on this approach in the discussion. A description of how the sensitivity and specificity analysis was done using the STATA diagti tool has been added under the statistical analysis section. The limitations of POC-CCA have been mentioned under the discussion section

There is insufficient information in the statistical methods section about the methods used in the nutritional section. For instance, the type of correlation used between BMI and MUAC needs to be specified; some results refer to F values for tests that aren’t specified, the inclusion of covariates in the model from Table S8 has not been justified, etc. Under the statistical analysis section, the last paragraphs have been rewritten to address the correlation analysis and linear regression analysis used.

**-Are there concerns about ethical or regulatory requirements being met?**
Yes. Consent was obtained on day 2. This means that investigators did not have consent to process the POC-CCA samples upon collection on day 1. Hence, the results of the participants that did not give consent on day 2 may need to be removed from the study. Indeed, consent and recruitment into the study was done on day2 following the screening using urine POC CCA on day 1. The POC-CCA screening was used for mapping and determining the prevalence of schistosomiasis as part of the Schistosomiasis National Control Program, for this no individual consent is required. The data to determine the infection intensity (CCA, KK, CAA) and nutrition status (Age, Weight, Height, Sex) presented in this study was from only those children who assented and whose parents consented to taking part in the study (Recruited participants, Figure 1B).

**Other concerns:**
Line 217: if the two technicians disagreed, how was this resolved? Disagreement with regards to scoring was resolved through taking the average readings from the independent technician readings, and this is what is indicated in the methodology.
Lines 211-223 and 227-233: if these lines are not relevant for this study, consider removing them. Community engagement through the community gatekeepers (teachers, political leaders etc) is described to emphasise that appropriate community engagement practices were followed (ethics) in human participant recruitment for the study. Lines 214-215 about cryopreserving samples deleted; line 221-223 on miracidia placement on FTA removed; line 227 – 233 on RBC lysis and Paxgene removed.

Reviewer #2: Yes

--------------------

**Results**

-Does the analysis presented match the analysis plan?
-Are the results clearly and completely presented?
-Are the figures (Tables, Images) of sufficient quality for clarity?


**Reviewer #1:**

**-Does the analysis presented match the analysis plan?**
There was no clear analysis plan. The analysis plan was mentioned in the last paragraph of the Introduction section indicating the aim, scope and parameters to be analysed in the study. The methodology and results were sectioned in a manner that addresses these objectives. Furthermore, the revised statistical section of the methods has been detailed to reflect the analysis plan.

**-Are the results clearly and completely presented?**
Not always.
Prevalence:
• Why was prevalence based on POC-CCA results only? This needs justifying. POC-CCA was used for screening all the individuals whereas not all the individuals where subjected to the KK and CAA tests. For field screening, CCA is more sensitive than KK (Kittur 2016) and this statement has been added to the section
• No extrapolation to district can be made (lines292-294). This statement and extrapolation to district has been removed
• Line 297: should it read “point” instead of “overall”? This has been corrected
• Lines 301-304: I am not certain that these results are needed. The results are needed for a statistical justification of observed point prevalence based on the Hierarchical sampling analysis for spatial sites.

Intensity of infection:
• Line 315: what does “confidence level 0.092” mean? This has been removed
• Line 322: what does “confidence level 3.981” mean? This has been removed
POC-CCA precision
• I suggest that this subheading be created. POC-CCA precision subheading has been created
• Line 359: expand on this: “The 6 KK positive cases that were negative by CAA did not greatly affect the specificity of the test”. How did the authors quantify this? This was in reference to the 88% specificity observed. We did not quantify this and therefore the statement has been removed.
• Table S3: To avoid confusion between POC-CCA and CCA, I suggest that every time POC-CCA is referred to, the authors use the prefix “POC”. POC-CCA has been added to the table
• Table S3: there should be a difference between “Controls CCA (=0)” when trace is considered positive and negative, whereas in Table S3, in both cases, FN is 2 and TN is 21. A reanalysis has been done and the correct values indicated
Nutrition status
• Line 362: Remove the word “impact” as this word implies a causal effect that was not assessed in this study. The word “impact” removed from sub heading
• Line 367: does the F-test result cited refer to BMI or MUAC? How was this test done? If it was ANOVA, then the statement “the girls of the same age group showed higher levels (F = 1.7, P=8.12e-08) of both BMI and MUAC than the boys” would be incorrect. ANOVA was carried out with the F test result and P-values showing a significant difference between the boys and girls. The statement has been changed to, “there was a significant difference between the girls and boys with respect to both the BMI (F = 1.7, P=8.12e-08) and MUAC (F=1.5, 4.9e-05) (Figure S4C&D).”
• Line 373: What are “sites with control individuals”? Should these sites have been mentioned in the methods section? Statement rephrased to “sites of Pamitu and Alwi with predominantly uninfected individuals”
• Line 385-386: this statement is misleading given the results presented in the next paragraph. This statement was meant to highlight the effect of cofounders in linear mixed models on observations that were made in line 385-386
Other recommendations:
• Always report p-values with r values. This has been addressed.

**-Are the figures (Tables, Images) of sufficient quality for clarity?**
On some graphs, the authors do not mention which estimate they are reporting, e.g. mean. We have checked and addressed the parameters on the graphs accordingly

The word “phenotype” has not been consistently removed. “Phenotype” has been removed

Reviewer #2: Yes

--------------------

**Conclusions**

-Are the conclusions supported by the data presented?
-Are the limitations of analysis clearly described?
-Do the authors discuss how these data can be helpful to advance our understanding of the topic under study?
-Is public health relevance addressed?


**Reviewer #1:**

**-Are the conclusions supported by the data presented?**
The conclusions are not fully supported by the data presented. 
• The authors do not fully explore the limitations of POC-CCA (they only focused on one of its limitations “misdiagnosis when calling trace results as positive”, but there are other relevant concerns); the authors do not explore the limitations of using CCA as a gold standard (particularly given that 6 KK + and CCA- cases were observed) In the discussion, we have included other limitations of POC-CCA such as the inability to detect *S. haematobium* and hence missing its prevalence in the population. In addition, even though the CAA was used as gold standard to determine the sensitivity and specificity of POCCCA and KK, its major limitation is the practicability in a field setting.
• The authors state that “… The impact of this is likely to be compounded by high levels of under nutrition and stunting especially among the boys”. However, this directly contradicts their finding that “we did not find any association between schistosomiasis infection and stunting” (line 483). The under nutrition and stunting observed in this study has also been reported in this region in the Uganda demographic health survey 2014 and is possibly due to numerous other factors (frequency of meals etc). We tested for association between the observed stunting and schistosomiasis and show that there is no relationship. We have clarified our meaning by changing this to :” The impact of the high prevalence of schistosomiasis on child health is likely to be compounded by high levels of under nutrition and stunting especially among the boys”
-Are the limitations of analysis clearly described?
The limitations of the analysis are insufficiently described. The authors only mention one limitation (lines 488-489). Other key limitations of the study have been included mainly resulting from the COVID19 pandemic which affected the recruitment for the survey.

Discussion
Line 458: this study was not designed to assess this statement. We did not do MDA assessment but the statement was meant to discussed the high prevalence despite the annual MDA and we have rewritten this sentence to make that clear.
Line 484: “we observed lower BMI levels in the schistosomiasis cases than controls” directly contradicts lines 369-371: “The average BMI for the S. mansoni infected cases was 17.09±2.04 and that of the uninfected controls was 15.5±2.39”. The statement has been rephrased as “But contrary to those studies, we did not find any association between schistosomiasis infection and stunting; even though we observed high levels of stunting (48%) in the study population.”

Abstract
Lines 44-46: “Efforts to control schistosomiasis infection in Uganda have been greatly affected by … impact of disease on child growth”. We still don’t know what impact the disease has on child growth, hence we cannot be certain that this is having an impact on the efforts to control schistosomiasis.

We have rewritten this sentence as follows: “. It is also important to understand the impact of infection on child growth and development in order to allocate appropriate resources and effort to the control of the disease. “

Line 62: this study was not set up to assess “attribution”. Replaced with “associated with”
Line 64-65: the statement that “high levels of stunting might have an influence on morbidity” is misleading, particularly given the results of this study. Rephrased statement as “We observed high levels of stunting but even though this was not associated with schistosomiasis, there is a need for improved nutrition among the children in the endemic area.”

Title
“High levels of infection”; infection was primarily light (lines 322-323). Replaced levels with Prevalence

Reviewer #2: Yes

--------------------

**Editorial and Data Presentation Modifications?**


Use this section for editorial suggestions as well as relatively minor modifications of existing data that would enhance clarity. If the only modifications needed are minor and/or editorial, you may wish to recommend “Minor Revision” or “Accept”. 

Reviewer #1: (No Response)

Reviewer #2: In some elements of the text, the sentence structure requires revision to enhance readability.

--------------------

**Summary and General Comments**


Use this section to provide overall comments, discuss strengths/weaknesses of the study, novelty, significance, general execution and scholarship. You may also include additional comments for the author, including concerns about dual publication, research ethics, or publication ethics. If requesting major revision, please articulate the new experiments that are needed.

Reviewer #1: Please refer to my extensive comments in the sections above

Reviewer #2: The authors have addressed reviewer comments.

--------------------

PLOS authors have the option to publish the peer review history of their article ([what does this mean?](https://journals.plos.org/plosntds/s/editorial-and-peer-review-process#loc-peer-review-history)). If published, this will include your full peer review and any attached files.


If you choose “no”, your identity will remain anonymous but your review may still be made public.


**Do you want your identity to be public for this peer review?** For information about this choice, including consent withdrawal, please see our [Privacy Policy](https://www.plos.org/privacy-policy).

Reviewer #1: No

Reviewer #2: No


Figure Files:
While revising your submission, please upload your figure files to the Preflight Analysis and Conversion Engine (PACE) digital diagnostic tool, https://pacev2.apexcovantage.com. PACE helps ensure that figures meet PLOS requirements. To use PACE, you must first register as a user. Then, login and navigate to the UPLOAD tab, where you will find detailed instructions on how to use the tool. If you encounter any issues or have any questions when using PACE, please email us at figures@plos.org.
 
Data Requirements:
Please note that, as a condition of publication, PLOS' data policy requires that you make available all data used to draw the conclusions outlined in your manuscript. Data must be deposited in an appropriate repository, included within the body of the manuscript, or uploaded as supporting information. This includes all numerical values that were used to generate graphs, histograms etc.. For an example see here: http://www.plosbiology.org/article/info%3Adoi%2F10.1371%2Fjournal.pbio.1001908#s5.
 
Reproducibility:
To enhance the reproducibility of your results, we recommend that you deposit your laboratory protocols in protocols.io, where a protocol can be assigned its own identifier (DOI) such that it can be cited independently in the future. Additionally, PLOS ONE offers an option to publish peer-reviewed clinical study protocols. Read more information on sharing protocols at https://plos.org/protocols?utm_medium=editorial-email&utm_source=authorletters&utm_campaign=protocols

__________________________________________________
In compliance with data protection regulations, you may request that we remove your personal registration details at any time. (Use the following URL: https://www.editorialmanager.com/pntd/login.asp?a=r). Please contact the publication office if you have any questions.
